# Exploring women’s sexuality during pregnancy : sociodemographic, culturel and relational characteristics

**DOI:** 10.1192/j.eurpsy.2023.2335

**Published:** 2023-07-19

**Authors:** R. Ben Soussia, R. Melki, H. Ben Sassi, W. Bouali, D. Toumi, L. Zarrouk

**Affiliations:** 1 Department of psychiatry, Hopital Universitaire Taher SFAR Mahdia, Mahdia; 2 Department of psychiatry, Hopital Universitaire Fattouma Bourguiba; 3 Family medicine, Faculté de Médecine de Monastir; 4 Department of Gynecology, Centre de maternité et de néonatologie Monastir, Monastir, Tunisia

## Abstract

**Introduction:**

Pregnancy is a period of important physical, psychological and hormonal changes. All these changes affect daily life, relationships with others, the relationship with the body and particularly the intimate and sexual aspect within the couple.

**Objectives:**

to describe the sociodemographic and clinical profile of pregnant women, explore their perceptions and practices regarding sexuality during an uncomplicated pregnancy and to assess the impact of pregnancy on the couple’s sexuality.

**Methods:**

This is a cross-sectional, descriptive study conducted among pregnant women followed at the outpatient prenatal and women in immediate postpartum who gave birth in the obstetrics department of EPS Tahar Sfar Mahdia, during a period of 6 months from 01 September 2019 to 28 February 2020. The evaluation of women’s sex life during pregnancy was conducted using a predefined questionnaire and the validated scale translated into Arabic “Arabic Female Sexual Function Index (ArFSFI)”.

**Results:**

A total of 110 patients were included. The average age of the patients was 30.2 +/- 4.98 years. In 60 % of the cases, the patients were from rural areas. More than half (55%) of our patients had a primary education and were housewives (64%). They were married for love in 59% and the marriage was arranged for 41% of the women. More than 2/3 of our patients (71%) reported a good marital relationship. In relation to the current pregnancy: the majority of patients (70%) were in immediate post partum, the pregnancy was desired (98.1%), well experienced (62%).

For most of the patients (58,2%), sexual intercourse was possible and without risk during the whole pregnancy, but some women thought about the risk of abortion or premature delivery (37.3%), the risk of metrorrhagia (10%) and the risk of infection (6.4%). Most patients (87.2%) reported a decrease in the frequency of sexual intercourse during pregnancy. Most women reported a decrease in desire (51.8%), and sexual satisfaction (55.6%) during their pregnancies. For partner sexuality, desire was stationary for 89% and sexual satisfaction was decreased in 61.1% of cases. Almost half of our patients (42.7%) talked about sexuality : to the husband in 74% of cases, followed by social networks in 58% of cases. The motivation for talking about sexuality was in the majority of cases (61.7%) secondary to a complication (metrorrhagia/ Threatned premature labor..).The mean FSFI total score was 25.3 ± 2.8. More than 2/3 of the patients (70%) had a score <26.55 indicating female sexual dysfunction.

**Conclusions:**

Performing sexual acts during pregnancy has very low risk, contraindications are rare and specific. It is therefore important that patients be informed and reassured on this subject. Information on sexuality during pregnancy should be systematically provided to women during their pregnancy follow-up.

**Disclosure of Interest:**

None Declared

